# Boosting the Faradaic Efficiency of Br^−^‐Mediated Photoelectrochemical Epoxidation by Local Acidity on α‐Fe_2_O_3_

**DOI:** 10.1002/advs.202401685

**Published:** 2024-04-25

**Authors:** Meng‐Yu Duan, Dao‐Jian Tang, Jie Yang, Si‐Peng Yang, Chao‐Yuan Deng, Yu‐Kun Zhao, Ji‐Kun Li, Yu‐Chao Zhang, Chun‐Cheng Chen, Jin‐Cai Zhao

**Affiliations:** ^1^ Key Laboratory of Photochemistry CAS Research/Education Center for Excellence in Molecular Sciences Institute of Chemistry Chinese Academy of Sciences Beijing 100190 P. R. China; ^2^ University of Chinese Academy of Sciences Beijing 100049 P. R. China; ^3^ Department of Chemistry National University of Singapore 12 Science Drive 2 Singapore 117549 Singapore

**Keywords:** α‐Fe_2_O_3_, alkenes epoxidation, Br^−^/HBrO‐mediated, high FE, local acidity

## Abstract

The redox mediated photoelectrochemical (PEC) or electrochemical (EC) alkene oxidation process is a promising method to produce high value‐added epoxides. However, due to the competitive reaction of water oxidation and overoxidation of the mediator, the utilization of the electricity is far below the ideal value, where the loss of epoxidation's faradaic efficiency (FE) is ≈50%. In this study, a Br^−^/HOBr‐mediated method is developed to achieve a near‐quantitative selectivity and ≈100% FE of styrene oxide on α‐Fe_2_O_3_, in which low concentration of Br^−^ as mediator and locally generated acidic micro‐environment work together to produce the higher active HOBr species. A variety of styrene derivatives are investigated with satisfied epoxidation performance. Based on the analysis of local pH‐dependent epoxidation FE and products distribution, the study further verified that HOBr serves as the true active mediator to generate the bromohydrin intermediate. It is believed that this strategy can greatly overcome the limitation of epoxidation FE to enable future industrial applications.

## Graphical abstract

A method of PEC Br^−^/HBrO mediated alkene epoxidation with a near‐quantitative selectivity and 100% FE is achieved by modulating Br^−^ concentration and the local acidity around the interface of α‐Fe_2_O_3_ photoanode, in which bromohydrin product is identified as the intermediate and transformed to epoxide with the removal of HBr when meeting OH^−^.

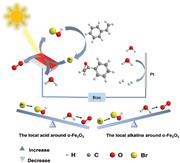

## 1 Introduction

Epoxides are widely‐used building blocks for the synthesis of functional polymers and pharmaceuticals.^[^ ^1^ ^]^ Due to its green, sustainable and safe characteristics,^[^ ^2^ ^]^ EC^[^ ^3^ ^]^ and PEC^[^ ^4^ ^]^ epoxidation of alkenes has been studied as an efficient route for the synthesis of epoxides. However, due to the hydrophobic nature of C═C bonds and the large steric hindrance of alkenes,^[^ ^5^ ^]^ coupling with the strong hydrophilic property of photoanodes,^[^ ^6^ ^]^ the direct PEC epoxidation exhibits relatively poor performance.^[^ ^7^ ^]^ The indirect PEC epoxidation reactions mediated by halogen ions such as chloride and bromide are reported to exhibit excellent selectivity.^[^ ^8^ ^]^ In these halide‐mediated epoxidations, the halide ions are initially oxidized to active intermediate species such as Cl_2_/Br_2_ and HOCl/HOBr, which react with alkenes in the bulk solution to realize epoxidation reaction effectively, overcoming the limits of mass transport and weakening interaction between alkenes and the oxide electrode. Nevertheless, a significant challenge that demands recognition is the occurrence of competitive reactions leading to a substantial loss in FE. In previous reported bromine‐mediated systems, only 65% FE of water‐soluble alkenes^[^ ^9^ ^]^ and 40–50% FE for most other alkenes were obtained.^[^ ^10^ ^]^ These competitive reactions encompass the oxidation of water to O_2_^[^ ^11^ ^]^ and the overoxidation of active halogen species to chlorate/bromate during halogen‐mediated epoxidation process.^[^ ^10^ ^]^ Therefore, how to address these competitive reactions is the key to boost the FE of epoxidation.

Recently, Leow et al reported an interesting chloride‐mediated electrochemical system for the production of ethylene and propylene epoxides, in which 70% FE can be achieved.^[^ ^8b^ ^]^ In this system, an anion exchange membrane was used to separate the anolyte and catholyte chambers. Active chlorine species (Cl_2_ and HOCl) produced at the anode effectively reacted with alkenes to produce chlorohydrin in the acidic anolyte chamber. Subsequently, ethylene oxide was generated from the reaction between the anodic chlorohydrin and the cathodic OH^−^ by mixing the catholyte and anolyte output streams. Li's group demonstrated a Br^−^/Br_2_‐mediated epoxidation system on BiVO_4_, in which water‐soluble styrene derivations can reach to 65% FE value.^[^ ^9^ ^]^ In our previous study, we reported a Br^−^/BrO^−^‐mediated epoxidation strategy by utilizing oxygen atom transfer property of α‐Fe_2_O_3_ photoanode.^[^ ^10^ ^]^ Although achieving excellent selectivity of alkenes, there is still a loss of ≈50% FE for most substrates at Br^−^ concentration of 100 mM. The lost FE is found to dominantly stem from the overoxidation of Br^−^ to inactive BrO_3_^−^.

In this study, we report that the FE can be markedly improved by reducing the concentration of Br^−^ and modulating the transportation of the H^+^ formed on the anode and the OH^−^ produced on the cathode in a one‐compartment cell. Further, by using a two‐compartment cell to eliminate the transportation of cathodic OH^−^ to neutralize the anodic H^+^, a near‐unit selectivity and ≈100% FE for the conversion of styrene into the corresponding epoxide can be obtained under ambient conditions. According to the experimental results, a Br^−^/HOBr mediated mechanism on α‐Fe_2_O_3_ is proposed: Br^−^ is oxidated to BrO^−^ through the pathway of oxygen atom transfer on α‐Fe_2_O_3_; HOBr species are formed under the local acidity around the interface of α‐Fe_2_O_3_ photoanode without stirring speed; Subsequently, HOBr species react with alkenes to generate bromohydrin, which can further conduct the dehydrobromination to epoxide. The local acidity around the interface of α‐Fe_2_O_3_ photoanode weakens sharply the competing reaction of water oxidation to O_2_ and contributes to the significant enhancement of FE.

## 2 Results

### 2.1 Epoxidation Optimization and Substrates Expansion

**Figure 1:**
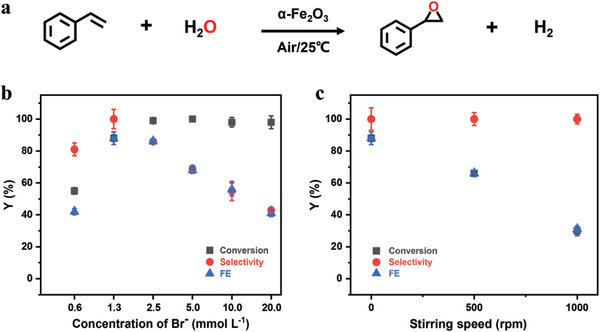
a) Reaction equation of Br^−^ mediated epoxidation for styrene substrate. b) The epoxidation performance for different Br^−^ concentrations (0.6–20 mm) at 0.34 V versus Fc/Fc^+^ with 5 m H_2_O and 5 mm styrene; c) The epoxidation performance for different stirring speeds (0–1000 rpm) at 0.34 V versus Fc/Fc^+^ with 5 m H_2_O, 1.3 mm Br^−^, and 5 mm styrene. Each error bar denotes the standard deviation of data from three experiments.

The Br^−^ mediated epoxidation reaction on α‐Fe_2_O_3_ photoanode was first investigated by using styrene as model alkene in a one‐compartment cell (Figure S1, Supporting Information, and detailed characterizations in Figure S2, Supporting Information), in which platinum wire and Ag/AgCl serve as the counter and reference electrodes, respectively. An acetonitrile solution with 5 M water and 100 mm tetrabutylammonium tetrafluoroborate (TBABF_4_) was used as the electrolyte. As shown in **Figure** **1**a,b, an examination on the effect of Br^−^ concentration shows that all the conversion, selectivity, and FE for the epoxidation reaction are quite low (53%, 77%, and 40%, respectively) at very low Br^−^ concentration (0.6 mm, the concentration of Br^−^ impurity in the employed TBABF_4_ electrolyte). When the Br^−^ concentration increased to 1.3 mm, the conversion, selectivity, and FE (88%, 100%, and 88%, respectively) are significantly improved at the same PEC coulomb (4.8 C, the theoretical charge for the complete transformation of alkene to its epoxidized product), indicative of the crucial role of Br^−^ in the PEC epoxidation of styrene. Over that concentration of Br^−^, however, both the selectivity and FE exhibited a rapid decrease with the increased Br^−^ amount due to the production of by‐product 1,2‐dibromo‐2‐phenylethane (Figure S3, Supporting Information). It is remarkable that only 1.3 mm of Br^−^ can achieve a FE value of 88%, which is far beyond the most current reported ones for the PEC or EC epoxidation reactions (detailed list see Table S1, Supporting Information).

With the mediation of low concentration of Br^−^, variety of styrene derivatives can be selectively oxidized into corresponding epoxides on α‐Fe_2_O_3_. As shown in **Table** **1** and Figures S4–S11 (Supporting Information), halogen‐ and other groups‐substituted substrates behaved the satisfied FE values and selectivity of epoxides. Concerning the halogen‐substituted substrates (entries 4, 5, 6 in Table 1), the epoxidation process maintained the impressive FE values and selectivity in the range of 80–90% and over 90%, respectively. Notably, the substrate β‐methyl styrene achieved 86% FE and 92% selectivity at a near‐complete conversion. However, when the para‐group constituted an electron‐donor‐methyl substitution, both the selectivity and FE exhibited a slight decrease. This trend was also observed in the reaction performance of the 2‐vinylenes substrate, where the FE and selectivity dropped to 70% and 80%, respectively. In summary, the system involving low concentration of Br^−^ demonstrated excellent adaptability across various styrene derivatives.

**Table 1:** The epoxidation performance of different styrene substituted alkenes on α‐Fe_2_O_3_.

Entry	Substrate^a)^	Conversion (%)^b)^	Selectivity (%)^c)^	FE (%)^d)^
1	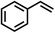	88 ± 2	100	88 ± 4
2^e)^	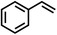	80	100	100
3^f)^	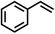	61	100	95
4	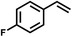	89	95	82
5	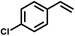	82	92	82
6	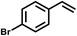	95	95	91
7	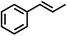	94	92	86
8	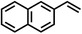	86	82	71
9	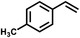	81	87	71
10	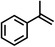	95	74	70

^a)^ Typical reaction conditions: 5 mL solution containing 5 mm substrates in CH_3_CN (5 M H_2_O) with 1.3 mM Br^−^ and 100 mm TBABF_4_, room temperature, applied potential 0.34 V versus Fc/Fc^+^. HPLC and ^1^H NMR were used to quantify the products;

^b)^ Conversion (%) is [(initial moles of substrate − moles of substrate at the end of reaction)/initial moles of substrate] × 100;

^c)^ Selectivity (%) is (the moles of epoxy product/the moles of substrate conversion) × 100;

^d)^ FE (%) is (the moles of epoxy product × 96485 × 2/the total charge of photoelectrolysis process) × 100;

^e)^ H Cell, 1 mm substrate and total solution 16 mL, 20 µL 1 m H_2_SO_4_ was added to the anode chamber;

^f)^ H Cell,1 mm substrate and total solution 16 mL.

A notable observation at the low concentration of Br^−^ is that the stirring speed presents a great influence on the performance of epoxidation. As shown in Figure 1c, although the selectivity for epoxidation remains quite high (≈99%) at different stirring speeds, both the conversion and FE gradually decreased with increasing stirring speed. The conversion and FE are ≈88% at 0 rpm and decrease to only ≈30% at 1000 rpm after the PEC coulomb of Q = 4.8 C. The decrease in conversion and FE value indicates that undesired reactions, besides epoxidation of styrene, become more competitive at a high stirring speed. In our previous study on the Br^−^ mediated epoxidation, we found that the loss in FE is mainly attributed to the overoxidation of Br^−^ to inactive BrO_3_^−^ at high Br^−^ concentration (100 mM).^[^ ^10^ ^]^ To further elucidate the impact of stirring speed on FE at low concentration of Br^−^, the mass balance of Br^−^ before and after the PEC reaction was analyzed using ion chromatography (IC). As depicted in Table S2 (Supporting Information), before the reaction, the concentration of Br^−^ is ≈1.24 mm (entry 1 in Table S2, Supporting Information), which becomes 1.06 mm after passing the PEC coulomb of 4.8 C at 0 rpm stirring speed. The corresponding concentration of BrO_3_^−^ is 0.16 mm after the reaction (entry 2 in Table S2, Supporting Information). Conversely, at 1000 rpm, 0.74 mm Br^−^ and 0.18 mm BrO_3_^−^ were detected after the PEC reaction with the same coulomb (entry 3 in Table S2, Supporting Information). The formation of BrO_3_^−^ accounts for 9% and 11% of the total coulomb at 0 and 1000 rpm, respectively (entries 2 and 3 in Table S2, Supporting Information). These results indicate that, although the overoxidation of Br^−^ is still the dominant origin of FE loss at 0 rpm (9% of 12%), it is not the main contributor of the FE loss at high stirring speed (11% of 70%). Instead, we propose that the competitive water oxidation reaction should be the main origin of lost FE at high stirring speed and low concentration of Br^−^ (see below for more evidence). Moreover, the sum of the detected Br^−^ and BrO_3_^−^ after the reaction at 0 rpm is 1.22 mM, close to the initial concentration 1.24 mm. The total concentration of Br^−^ and BrO_3_^−^ is only 0.92 mm at 1000 rpm, which is significantly lower than that at 0 rpm and the initial Br‐ concentration. These results mean that other bromine species besides BrO_3_^−^ may be generated at high stirring speed.

### 2.2 PEC Behaviors and Intermediate Species

**Figure 2:**
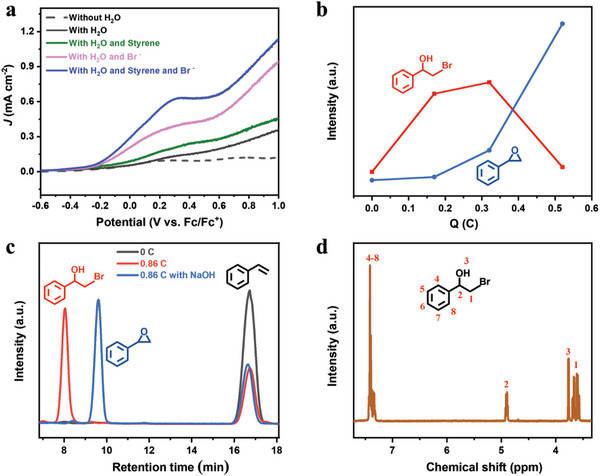
a) LSVs of the α‐Fe_2_O_3_ photoanode in the 100 mm TBABF_4_ acetonitrile solution with (solid) or without (dash) water under different conditions. The concentrations of styrene and Br^−^ were 5 and 1.3 mm, respectively. Scan rate, 50 mV s^−1^; b) The changes of the epoxidation products between epoxide and bromohydrin with different photoelectrolysis coulomb; c) The HPLC spectra of bromohydrin of 1 mm styrene in a two‐compartment cell at 0.34 V versus Fc/Fc^+^ on α‐Fe_2_O_3_, and the peak of epoxide obtained with adding NaOH to the solution of after the reaction; d) ^1^H NMR spectra of bromohydrin product.

To further understand the system of low concentration Br^−^ mediated styrene epoxidation, the linear scanning voltammetry (LSV) curves were also studied. As shown in **Figures** **2**a and Figure S12 (Supporting Information) tetrabutylammonium tetrafluoroborate (TBABF_4_) as the electrolyte. The addition of 5 mm styrene only enhanced the photocurrent to a quite limited extent, which indicates that the direct oxidation of styrene is quite inefficient on α‐Fe_2_O_3_. However, when 1.3 mm of Br^−^ was added, photocurrent is noticeably increased. Moreover, the introduce of Br^−^ resulted in a negative shift (≈200 mV) of the onset potential, which suggests that the oxidation of Br^−^ is prior to water oxidation under the present conditions, consistent with the previous report.^[^ ^10^ ^]^ Interestingly, the photocurrent increase caused by the addition of styrene in the presence of Br^−^ was much larger than that in the absence of Br^−^, implying that the presence of Br^−^ would greatly enhance the oxidation of styrene. Such a result in the presence of low concentration of Br^−^ is different with that of high concentration bromide‐mediated system, where the addition of alkenes hardly changed the photocurrent.^[^ ^10^ ^]^ A notable characteristic in the LSV curves is that an evident oxidative peak appears in the Br^−^ alone or particularly the Br^−^ and styrene system. This peak should be attributed to the limited mass transport of Br^−^ via diffusion from the bulk solution to the surface of the photoanode. To support this, we conducted the LSV experiments on the α‐Fe_2_O_3_ photoanode by using different Br^−^ concentrations. As shown in Figure S13 (Supporting Information), the peak disappears gradually with the concentration of Br^−^ increasing to 10 mm, where the Br^−^ diffusion is not limited anymore because Br^−^ is sufficient on the surface of the α‐Fe_2_O_3_ photoanode. These observations suggest that, at low Br^−^ concentration, the mass transport may be an important factor to Br^−^ mediated oxidation reaction of styrene without stirring.

**Scheme 1:**
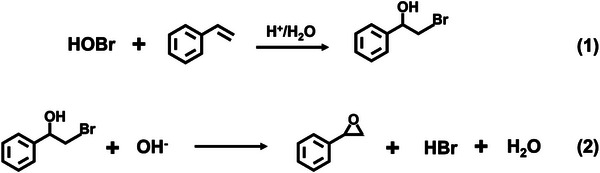
The reaction scheme of styrene epoxidation.

The intermediate of styrene epoxidation was detected by the high‐performance liquid chromatography (HPLC). As shown in Figure S14 (Supporting Information), during the PEC oxidation of styrene without stirring, besides the peak from the epoxidized product (with retention time of 9.6 min), a peak with retention time of 8 min was observed, which is identified to bromohydrin product of styrene (by ^1^H NMR Figure 2d). The amount of this bromohydrin is gradually increased at the primary stage of the PEC reaction and reaches to its maximum at PEC reaction of 0.32 C. After that, a rapid decrease is observed with prolonged reaction time, while the formation of styrene epoxide is accelerated during the whole PEC process (Figure 2b; Figure S14, Supporting Information). Such a changing profile of products is a typical indication that bromohydrin product is the intermediate for the epoxide formation. It is evident that the bromohydrin intermediate is first formed at the early stage, and then transformed into epoxide with extended reaction time. It is known that, under acidic conditions, the active bromine species should exist in the form of hypobromous acid (HOBr). As a powerful electrophile reagent,^[^ ^12^ ^]^ HOBr can facilely oxidize the styrene to its bromohydrin products by electrophilic addition on the photoanode surface (Equation (1) in **Scheme** **1**). On the other hand, the basic condition is required for dehydrobromination of bromohydrin to epoxide (Equation (2) in Scheme 1). During the PEC reaction in a one‐compartment cell without stirring, the local pH at the photoanode and cathode would decrease and increase, leading to the accumulation of H^+^ and OH^−^, respectively. The OH^−^ diffuses from the cathode to the anode, and the formed bromohydrin on the photoanode migrates to the cathode at the same time. The OH^−^ and bromohydrin meet each other in the bulk electrolyte to achieve the dehydrobromination reaction (Equation (2) in Scheme 1). The acidic environment near the photoanode is maintained, which ensures the continuous formation of bromohydrins. However, under stirring conditions, H^+^ formed on the photoanode rapidly is neutralized by the OH^−^, so the acidity near the photoanode cannot be preserved. To check whether the cathodically‐produced OH^−^ is sufficient for the bromohydrin transformation, we carried out the photoelectrolytic experiment by using a two‐compartment cell (Figure S1, Supporting Information), in which a ceramic core between two compartments is used to suppress the massive exchange of electrolytes between them. 1 mm of bromohydrin was added to the cathodic cell. After passing a theoretical charge for oxidation of 1 mM alkene (1.55 C), all the bromohydrin in the cathodic cell is quantitively converted to epoxide (Figure S15, Supporting Information), which indicates that the OH^−^ produced on the cathodic electrode is sufficient to transform the corresponding amount of bromohydrin into its epoxide.

**Figure 3:**
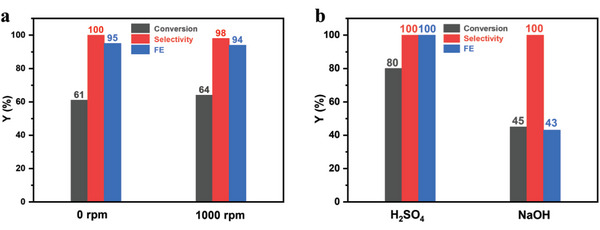
The epoxidation performance with different stirring speeds a) and different pH b) in the two‐compartment cell of 1 mM styrene with 100 mm TBABF_4_, 1.3 mm Br^−^, and 16 mL total solution at 0.34 V versus Fc/Fc^+^ on α‐Fe_2_O_3_.

To further verify the effect of local acidity and basicity in Br^−^ mediated epoxidation, the two‐compartment cell was further employed. As depicted in Figure 2c (red line), only bromohydrin was detected in the anode compartment, and its transformation to epoxide hardly occurred. The separated champers hinders the OH^−^ transportation to the anode compartment and consequently suppresses the dehydrobromination of bromohydrin. When the additional OH^−^ was added into the anodic solution, however, the bromohydrin would immediately transform into epoxide (Figure 2c). These results confirm that bromohydrin is formed first and then transformed to epoxide by the cathodically‐formed OH^−^. It is notable that the FE in two‐compartment cell can reach to 95%, even higher than that (88 ± 4%) in the one‐compartment cell. In addition, the stirring speed in this two‐compartment cell does not influence the conversion and FE values anymore (**Figure** **3**a). The nine cycle experiment results of the photoelectrolytic styrene epoxide show that the α‐Fe_2_O_3_ photoanode has good photoelectrochemical stability during the PEC reaction (Figure S16, Supporting Information). Moreover, when 20 µL of 1 m H_2_SO_4_ was added into the anodic compartment, where the concentration of H^+^ is ≈5 mm. Under this condition, the alkene is completely converted to bromohydrin after the PEC reaction. The formed bromohydrin was also quantitively transformed to epoxide by adding NaOH to basify the solution in the anodic cell. The left blue Bar in Figure 3b illustrates that the FE for the measured epoxide is almost 100%. By contrast, the addition of 20 µL of 5 m NaOH (12.5 mm OH^−^ in the reaction solution) was found to significantly decrease the FE value (43%) for epoxide formation (Figure 3b). All these results verify that the high FE value of epoxidation on α‐Fe_2_O_3_ without any stirring is attributed to the local acidity preservation around the photoanode surface, which favors the activation of Br^−^ and oxidation of styrene.

It is also remarkable that, on BiVO_4_ and TiO_2_ photoanodes (detailed characterizations in Figures S17 and S18, Supporting Information), different PEC behaviors for the Br^−^ mediated oxidation of styrene are observed from that on α‐Fe_2_O_3_. Under otherwise identical conditions, only 30% and 4% of the consumed styrene are transformed in styrene epoxide on BiVO_4_ and TiO_2_ photoanodes, respectively, and the corresponding FE value is 11% and 1% (Figure S19, Supporting Information), which is much lower than that on α‐Fe_2_O_3_ (99% of selectivity and 88% of FE). It has been reported that the surface trapped holes on α‐Fe_2_O_3_ photoanodes have been identified as high‐valent iron‐oxo intermediates (Fe^IV^ = O),^[^ ^13^ ^]^ which tends to oxidize Br^−^ to BrO^−^ via the oxygen atom transfer pathway, rather than to Br^•^ by a single electron transfer pathway.^[^ ^14^ ^]^ In contrast, the oxidation of Br^−^ on BiVO_4_ and TiO_2_ photoanodes prefers to proceed via an electron transfer pathway and lead to the formation of Br^•^. Such a pathway can also lead to the oxidation of water to OH^•^ particularly at low Br^−^ concentration and basic solution. Accordingly, the competition of water oxidation for photogenerated hole would greatly lower the FE value for styrene oxidation. In addition, the selectivity for epoxide on BiVO_4_ and TiO_2_ photoanodes is much lower (30% and 4%, respectively) than that on α‐Fe_2_O_3_, and accordingly great amounts of by‐products such as 1,2‐dibromo‐2‐phenylethane are detected (Figure S19a,b, Supporting Information). The formation of 1,2‐dibromo‐2‐phenylethane confirms that Br^•^ and then Br_2_ are formed in BiVO_4_ and TiO_2_ systems. By contrast, the selectivity for epoxide is quite high (≈100%) and hardly any dibrominated product is formed on α‐Fe_2_O_3_ under otherwise identical conditions (Figure S19c, Supporting Information), suggesting that no Br_2_ is involved in the formation of bromohydrin in the α‐Fe_2_O_3_ system. Furthermore, we also investigated the reaction between styrene and Br_2_ by adding Br_2_ into CH_3_CN solution with 5 m of water at different pHs. Confirmatively as shown in Figure S20 (Supporting Information), 1,2‐dibromo‐2‐phenylethane is formed regardless of the acidity and basicity.

The effect of water content on the epoxidation was also investigated. As depicted in Figure S21 (Supporting Information), the FE values and selectivity of epoxide product initially increased with the water content after the same photoelectrolysis coulomb (≈4.8 C), and a maximum FE was reached at 5 m of water. When water content increased from 5 to 10 m, the epoxidation performance remained nearly unchanged. However, exceeding 10 m of water would lead to reduced solubility of alkenes, resulting in a slightly turbid electrolyte or even phase separation, and consequently diminishing the FE and selectivity. At a lower water concentration (1.25 m H_2_O), several by‐products such as 1,2‐dibromo‐2‐phenylethane (retention time: 17.8 min) were detected (Figure S22, Supporting Information), which is irrelevant to the oxygen of reaction atmosphere (Figure S23, Supporting Information). The concentrations of Br^−^ and BrO_3_^−^ were also measured at different water content by IC (Table S3, Supporting Information). With the increase of water content, the concentrations of Br^−^ and BrO_3_^−^ gradually increased at the same PEC coulomb. For example, the total amount of Br^−^ (0.56 mm) and BrO_3_^−^ (0.06 mm) is only 0.62 mm after the PEC reaction in the presence of 1.25 m of H_2_O (entry 4 in Table S3, Supporting Information), which means that about half of Br^−^ is missed in this process. Confirmatively, after debrominating the dibrominated species by adding 5 m of NaOH and by transforming Br_2_ species into Br^−^ by 30 wt.% H_2_O_2_, the recovery of bromine by Br^−^ can reach to ≈82% (Figure S24, Supporting Information). It is reasonable that the reaction of formed Br^•^ radical or Br_2_ would lead to the formation of 1,2‐dibromo‐2‐phenylethane products when water is insufficient. By contrast, in the presence of sufficient water, HOBr is formed by an oxygen atom transfer pathway, which could react with styrene to bromohydrin.

## 3 Discussion

During the Br^−^ mediated oxidation of alkene, the first step is the oxidation of Br^−^ to active bromine species. As reported in our previous studies, the activation of Br^−^ on α‐Fe_2_O_3_ tends to occur in an oxygen atom transfer pathway, in which the oxygen atom in Fe^IV^ = O sites is transferred to Br^−^, and leads to the formation of BrO^−^.^[^ ^10^ ^]^ Such a non‐radical activation process is essential to the selectivity and the FE of epoxidation, as implied by poor performance of the reaction on radical‐based BiVO_4_ and TiO_2_ photoanodes (Figure S19a,b, Supporting Information). At high concentration of Br^−^, the formed HOBr species tends to take place disproportionation reaction and produces inactive BrO_3_^−^. Such overoxidation reaction of Br^−^ was found to the main origin of the FE loss for Br^−^ mediated epoxidation.^[^ ^10^ ^]^ It is evident that the disproportionation reaction between HOBr species would be largely avoided at low concentration of Br^−^. Accordingly, the low concentration of Br^−^ favors the high FE by hindering the over‐oxidation of Br^−^. However, besides the over‐oxidation of Br^−^, there is another origin for the loss of FE: the competitive water oxidation reaction.^[^ ^11^ ^]^ Such origin would become prominent at low concentration of Br^−^.

**Figure 4:**
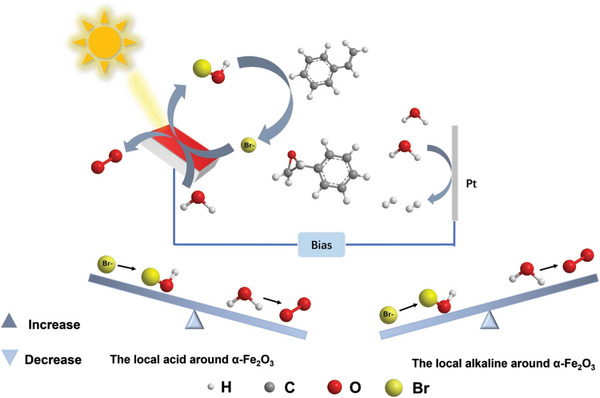
The proposed epoxidation mechanism of styrene in the low concentration Br^−^ mediated system on α‐Fe_2_O_3_.

It is well known that the water oxidation is highly pH‐dependent.^[^ ^15^ ^]^ Under acidic conditions, water oxidation would be suppressed, and accordingly the efficiency for the activation of Br^−^ would be enhanced relatively (**Figure** **4**). Moreover, the oxidation capability of HOBr should be much higher than that its anion BrO^−^. In acidic media, HOBr should be the dominant form of BrO^−^ species. Stirring, which enhances the mass transformation of anodic H^+^ and cathodic OH^−^, would decrease the local H^+^ concentration around the photoanode interface by the neutralizing reaction of H^+^ and OH^−^. By contrast, without string, the local H^+^ concentration is preserved due to the diffusion limitation of the H^+^ and OH^−^. Therefore, depressing the massive transportation of anodic and cathodic electrolytes by lowering string speed or using a two‐compartment cell, would largely enhance the FE value of epoxidation.

## 4 Conclusion

In summary, we have explored a low concentration Br^−^ mediated PEC system to achieve effective alkene epoxidation with water as the oxygen source and ≈95% FE of hydrogen evolution (Figures S25 and S26, Supporting Information). A variety of regulations on reaction conditions demonstrated that the interface local acidity on α‐Fe_2_O_3_ plays an important role in promoting epoxidation reaction. In this system, 1.3 mm Br^−^ serves as a true mediator to recycle the circle, which achieves an excellent epoxidation performance of styrene and its derivatives. A near‐quantitative selectivity and ≈100% FE of styrene oxide was obtained in ambient conditions, in which HOBr acts as active species on the photoanodic surface to trigger the formation of bromohydrin and then bromohydrin is converted into epoxide with the removal of HBr when meeting the free diffusing OH^−^. This study provides a promising method for the synthesis of epoxides with low concentration of bromine as the mediator and proposes a new sight to understand the significance of the interfacial local environment on α‐Fe_2_O_3_.

## 5 Experimental Section

All chemicals were purchased and used without further purification, except noted.

### Photoanode Preparation

The α‐Fe_2_O_3_ photoanode was fabricated by a two‐step method based on the literature that was reported earlier.^[^ ^16^ ^]^ First, NaNO_3_ (0.85 g, 0.1 m) and FeCl_3_ (2.43 g, 0.15 m) were added to 100 mL of deionized water, which was stirred intensely. Second, a piece of clean FTO (14Ω cm, 2 × 2 cm^2^) glass was placed to the reaction kettle (20 mL) and then the 10 mL obtained solution was transferred into the kettle. Then, FeOOH film was prepared by a hydrothermal method of 95 °C for 4 h. The α‐Fe_2_O_3_ photoanode was obtained through calcination of FeOOH film at 550°C for 2 h and subsequently annealed at 750 °C for 15 min.

The TiO_2_ photoanode^[^ ^11^ ^]^ was fabricated by adding titanium n‐butoxide (0.8 mL) into a HCl solution (30 mL of 35–37% HCl and 30 mL of deionized water) and stirring for 30 min. Then, using a hydrothermal method (150 °C for 4 h) to synthesize the precursor by transferring the above solutions and FTO (14Ω cm, 2 × 2 cm^2^) glass to a Teflon reactor. The obtained precursor was washed with deionized water and annealed in a muffle furnace at 450 °C for 1 h.

The BiVO_4_ photoanode was fabricated by a method of the literature that was reported previously.^[^ ^17^ ^]^ First, solution A: KI (3.32 g, 0.4 m) and Bi(NO_3_)_3_ (0.9701 g, 0.04 m) were added to 50 mL of deionized water, with pH adjusted to 1.7 by adding HNO_3_. The solution B: benzoquinone (0.4972 g, 0.23 m) was added to 20 mL ethanol solution. Solution C was obtained by mixing solutions A and B with stirring intensely for 20 min. Then, BiOI was deposited on a clean FTO (14Ω cm, 2 × 2 cm^2^) for 0.6 C in solution C, where the applied bias was −0.1 V versus Ag/AgCl and with a three‐electrode system. Subsequently, the obtained BiOI precursor was washed with deionized water and dried in the air. The BiOI was calcined at 450 °C for 2 h in a muffle furnace after dripping 0.4 mL VO(acac)_2_ dimethyl sulfoxide solution (1.0606 g, 0.4 m) onto the surface of BiOI. Finally, the obtained photoanode was soaked in 1 m NaOH to remove the residual V_2_O_5_ on its surface, which was followed by washing with deionized water and drying in air.

### Photoelectrochemical Study

A series of photoelectrochemical (PEC) experiments were performed on a CHI‐760E electrochemical (EC) workstation (Shanghai Chen hua Instrument Co, LTD). The 100 mW cm^−2^ of reaction light source was offered by a 300 W Xe lamp (Microsolar300, Beijing Perfectlight) with an AM 1.5G filter, a GRB3 (infrared filter), and a 50% attenuation film. A single‐compartment cell or double‐compartment cell was used as reaction chamber under ambient conditions. A α‐Fe_2_O_3_ photoanode (20 mm × 10 mm) was used as working electrode, and a platinum wire and a KCl saturated Ag/AgCl (3.5 M KCl leak‐free and 2 mm diameter, Innovative Instruments) electrode were used as the counter and reference electrodes, respectively. Linear sweep voltammetry experiments were conducted at a scan rate of 50 mV s^−1^. The PEC epoxidation of styrene was taking place in CH_3_CN with 5 m H_2_O and 1.3 mm Br^−^ (5 mL), using 100 mm TBABF_4_ as the electrolyte. The products and reactants were quantified by high performance liquid chromatography (HPLC). The potential of 0.34 V versus Fc/Fc^+^ (except special statement) was applied with photoelectrolysis experiments, which were conducted under 0 rpm and 25 °C. Using a 5 mm ferrocene/ferrocenium redox couple calibrated the potentials versus Ag/AgCl. According to Figure S27 (Supporting Information), (E (vs Fc/Fc^+^) = E (vs Ag/AgCl) – 0.46 V).

### Product Analysis

The sample of styrene and oxidative products were determined and quantified by an Agilent HPLC1260 system with a Dikma Diamond C‐18(2) column (250 × 3.0 mm, 5 µm film thickness). The mobile phase was acetonitrile (70%) and water (30%) with a flow rate of 0.2 mL min^−1^ and the detection wavelength of 216 nm. The temperature of the column was 30 °C and the injection volume of the sample was 20 µL. The retention time of the product peak was compared with the standard solution peak of the possible product (benzaldehyde, 1,2‐dibromo‐2‐phenylethane, bromohydrin et.al), and combining with NMR the products was determined finally. The concentration of styrene and oxidation products was conducted according to the corresponding standard curve of substance.

A 400 MHz Bruker instrument was used to detect the ^1^H NMR spectra of the corresponding epoxides generated in the PEC epoxidation. The residual solvent peaks (CD_3_CN) were as reference to calibrate the chemical shifts. To quantify the epoxides, an internal standard of 1,3,5‐trimethoxybenzene was added, which is stable in the system.

An ion chromatography (Thermo Fisher, ICS‐900) equipped with a Thermo Dionex IonPacTM AS18 anion exchange column (250 mm × 4 mm) and Reagent‐FreeTM Controller (Thermo Fisher, RFC30) was used to detect the ions concentration. The concentration of KOH eluents was held at 10 mm for 40 min. The concentration of Br^−^ and BrO_3_^−^ were analyzed according to the corresponding standard curve of different ions.

The H_2_^18^O (97% atom ^18^O) isotopic labelling experiments during the epoxidation of styrene were detected on an Agilent GC (7890B)‐MS (5977A) system. The GC condition for styrene epoxide was: 50 °C (3 min), 20 °C min^−1^, 170 °C (0 min), 10 °C min^−1^ and 280 °C (2 min) on a HP‐5MS column (30 m × 250 µm × 0.25 µm). Injection and detector temperatures were 180 and 300 °C, respectively.

### Calculation of FE for Epoxide Production and Hydrogen Evolution

The PEC experiments were conducted in a constant potential mode. The FEs of epoxides or bromohydrin were calculated through the following equation.

(1) $$\mathrm{FE}\;\left(\%\right)=\frac{n\times m\times F}{Q}\;\times 100\%$$

where *n* represents the number of electrons required for epoxidation of one alkene molecule (*n* = 2). *m* represents the number of moles of alkenes that have undergone epoxidation, *F* is Faraday's constant (96485 C mol^−1^), and *Q* represents the past total charge during photoelectrolysis. The FE of hydrogen was calculated by the same equation, where *m* represents the number of moles for produced hydrogen and *n* represents the number of electrons required for the reduction of one water molecule (*z* = 2).

### Photoanode Characterization

Transmission electron microscopy (TEM) and scanning electron microscopy (SEM) images were conducted using a HT770 (Hitachi, Japan), and SU8010 (Hitachi, Japan) respectively. X‐ray diffraction (XRD) spectra were collected on an X‐ray diffractometer (Empyrean, PANalytical) by using Cu‐Kα radiation. UV–vis spectra were taken with a UV–vis Hitachi U‐3900 spectrophotometer.

## Conflict of Interest

The authors declare no conflict of interest.

## Supporting information

Supporting Information

## Data Availability

The data that support the findings of this study are available in the supplementary material of this article.

## References

[1] a) K. Zhang , O. K. Farha , J. T. Hupp , S. T. Nguyen , ACS Catal. 2015, 5, 4859;

[2] a) B. Jiang , Y. Guo , F. Sun , S. Wang , Y. Kang , X. Xu , J. Zhao , J. You , M. Eguchi , Y. Yamauchi , H. Li , ACS Nano 2023,17, 13017; 37367960 10.1021/acsnano.3c01380

[3] a) K. Rossen , R. P. Volante , P. J. Reider , Tetrahedron Lett. 1997, 38, 777;

[4] a) Z. Masoumi , M. Tayebi , Q. Zaib , S. A. M. Lari , B. Seo , C.‐S. Lim , S. Yu , H.‐G. Kim , D. Kyung , Coord. Chem. Rev. 2024, 503, 215641;

[5] a) J. Yang , Y. Zhao , M. Duan , C. Deng , Y. Zhang , Y. Lei , J. Li , W. Song , C. Chen , J. Zhao , Energy Environ. Sci. 2024, 17, 183;

[6] F. Dorchies , A. Serva , D. Crevel , J. De Freitas , N. Kostopoulos , M. Robert , O. Sel , M. Salanne , A. Grimaud , J. Am. Chem. Soc. 2022, 144, 22734. 36468903 10.1021/jacs.2c10764

[7] a) X. Lin , Z. Y. Zhou , Q. Y. Li , D. Xu , S. Y. Xia , B. L. Leng , G. Y. Zhai , S. N. Zhang , L. H. Sun , G. H. Zhao , J. S. Chen , X. H. Li , Angew. Chem., Int. Ed. 2022, 61, 202207108; 10.1002/anie.202207108 35789523

[8] a) M. Chung , K. Jin , J. S. Zeng , K. Manthiram , ACS Catal. 2020, 10, 14015;

[9] X. Liu , Z. Chen , S. Xu , G. Liu , Y. Zhu , X. Yu , L. Sun , F. Li , J. Am. Chem. Soc. 2022, 144, 19770. 36260532 10.1021/jacs.2c06273

[10] Y. Zhao , M. Duan , C. Deng , J. Yang , S. Yang , Y. Zhang , H. Sheng , Y. Li , C. Chen , J. Zhao , Nat. Commun. 2023, 14, 1943. 37029125 10.1038/s41467-023-37620-8 PMC10082182

[11] Y. Zhao , C. Deng , D. Tang , L. Ding , Y. Zhang , H. Sheng , H. Ji , W. Song , W. Ma , C. Chen , J. Zhao , Nat. Catal. 2021, 4, 684.

[12] a) V. F. Ximenes , N. H. Morgon , A. R. de Souza , J. Inorg. 2015,146, 61; 10.1016/j.jinorgbio.2015.02.014 25771434

[13] a) O. Zandi , T. W. Hamann , Nat. Chem. 2016, 8, 778; 27442283 10.1038/nchem.2557

[14] a) Q. Guo , C. Y. Zhou , Z. B. Ma , X. M. Yang , Adv. Mater. 2019, 31, 1901997; 10.1002/adma.201901997 31423680

[15] a) L.‐F. Shen , B.‐A. Lu , Y.‐Y. Li , J. Liu , Z.‐C. Huang‐fu , H. Peng , J.‐Y. Ye , X.‐M. Qu , J.‐M. Zhang , G. Li , W.‐B. Cai , Y.‐X. Jiang , S.‐G. Sun , Angew. Chem., Int. Ed. 2020, 59, 22397; 10.1002/anie.202007567 32893447

[16] Y. Ling , G. Wang , D. A. Wheeler , J. Z. Zhang , Y. Li , Nano Lett. 2011, 11, 2119. 21476581 10.1021/nl200708y

[17] T. W. Kim , K.‐S. Choi , Science 2014, 343, 990. 24526312 10.1126/science.1246913

