# Supplementary material for: Boosting the Faradaic Efficiency of Br−‐Mediated Photoelectrochemical Epoxidation by Local Acidity on α‐Fe2O3
Source: Adv Sci (Weinh). 2024 Apr 25;11(25):2401685. doi: 10.1002/advs.202401685 (PMC11220633; doi:10.1002/advs.202401685)
Supplement: Supplementary file 1 — Supporting Information [file ADVS-11-2401685-s001.pdf]

ADVANCED SCIENCE
Open Access

Supporting Information

for *Adv. Sci.*, DOI 10.1002/advs.202401685

Boosting the Faradaic Efficiency of Br^{-}-Mediated Photoelectrochemical Epoxidation by Local Acidity on α-Fe_2O_3

Meng-Yu Duan, Dao-Jian Tang, Jie Yang, Si-Peng Yang, Chao-Yuan Deng, Yu-Kun Zhao, Ji-Kun Li, Yu-Chao Zhang*, Chun-Cheng Chen* and Jin-Cai Zhao*

Supporting Information

Boosting the faradaic efficiency of Br^--mediated photoelectrochemical epoxidation by local acidity on α-Fe_2O_3

Meng-Yu Duan[+], *Dao-Jian Tang*[+], *Jie Yang, Si-Peng Yang, Chao-Yuan Deng, Yu-Kun Zhao*, Ji-Kun Li, Yu-Chao Zhang*, Chun-Cheng Chen*, and Jin-Cai Zhao*

[+] These authors contributed equally to this work.

M.-Y. Duan, D.-J. Tang, J. Yang, S.-P. Yang, C.-Y. Deng, Prof. J.-K Li, Prof. Y.-C Zhang, Prof. C.-C. Chen, and Prof. J.-C. Zhao

Key Laboratory of Photochemistry, CAS Research/Education Center for Excellence in Molecular Sciences, Institute of Chemistry, Chinese Academy of Sciences, Beijing, P. R. China

E-mail: ccchen@iccas.ac.cn

yczhang@iccas.ac.cn

M.-Y. Duan, D.-J. Tang, J. Yang, S.-P. Yang, C.-Y. Deng, Prof. J.-K Li, Prof. Y.-C Zhang, Prof. C.-C. Chen, and Prof. J.-C. Zhao

University of Chinese Academy of Sciences, Beijing, P. R. China

Yu-Kun Zhao

Department of Chemistry, National University of Singapore, Singapore, 12 Science Drive 2, Singapore, 117549

E-mail: y.zhao@nus.edu.sg

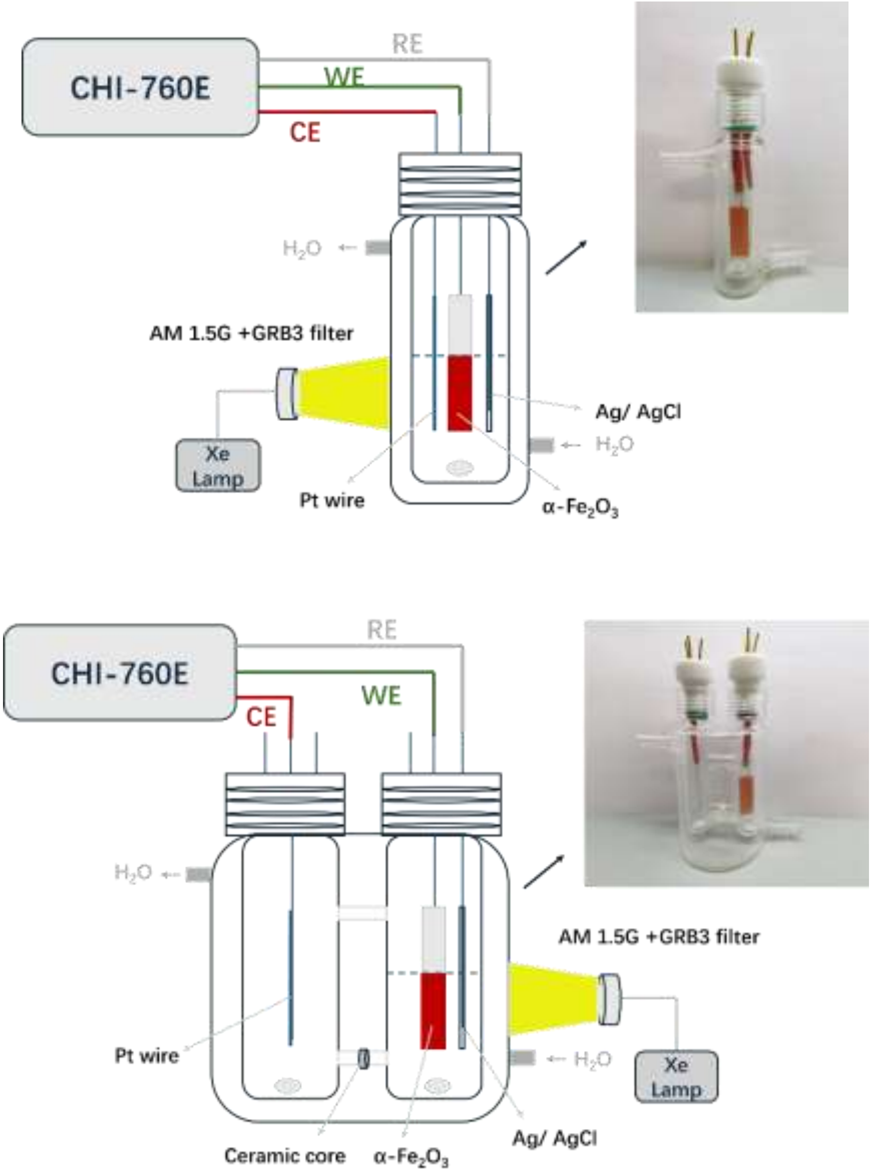

Figure S1. The setup of PEC cell. The effective surface area of the α-Fe_2O_3 photoanode was 1×2 cm^2, and the total volume of the reaction solution in the one-compartment cell and the two-compartment cell was 5 mL and 16 mL, respectively. In the PEC epoxidation reactions, the applied bias was 0.34 V vs. Fc/Fc^+ and the substrate concentration was 5 mM and 1 mM in one-compartment cell and two-compartment cell respectively with 100 mM $TBABF_4$ and 1.3 mM Br^- in the Air atmosphere at 25 °C

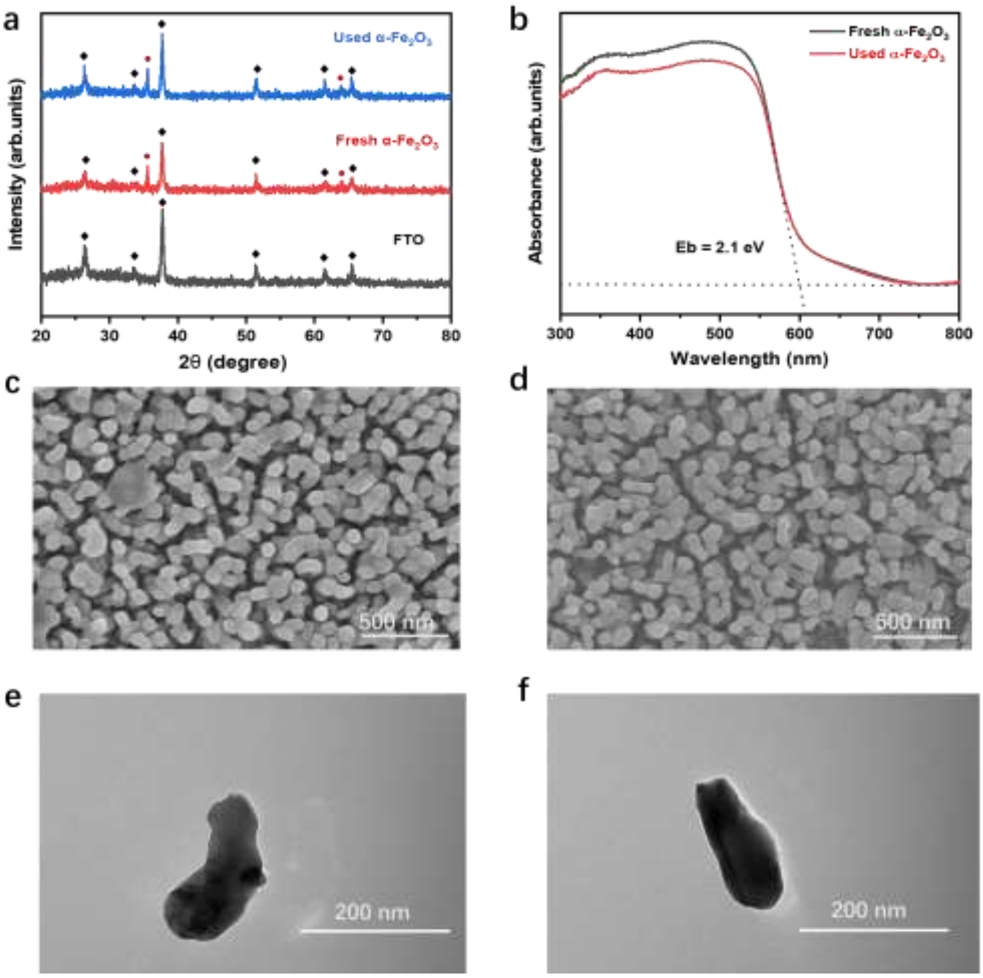

Figure S2. The characterizations of fresh and used α-Fe_2O_3 photoanodes. a) XRD spectra; b) UV-vis diffuse spectra; SEM images of fresh c) and used d) α-Fe_2O_3; TEM images of fresh e) and used f) α-Fe_2O_3.

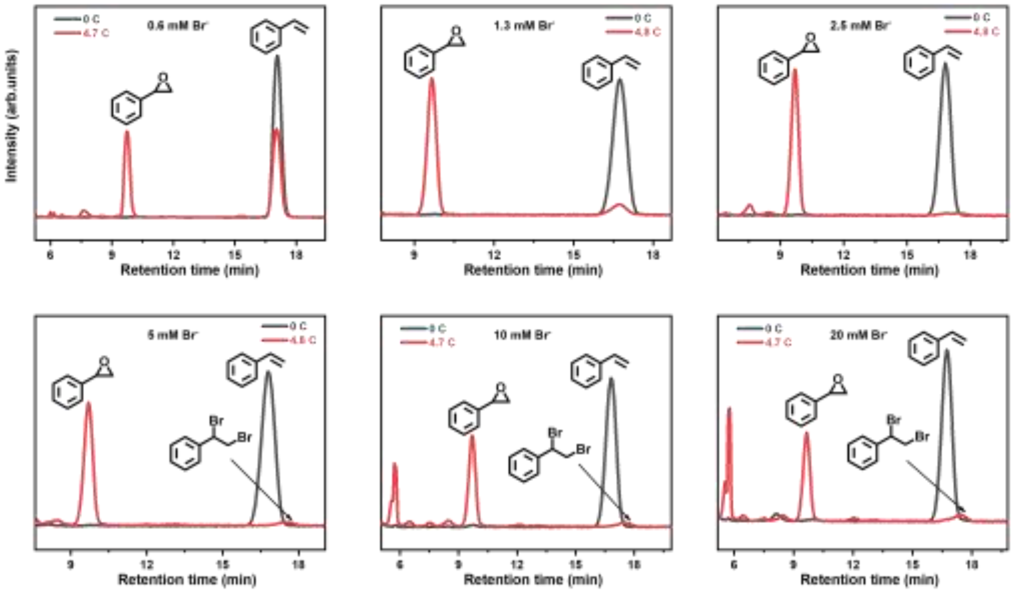

Figure S3. The HPLC spectra of styrene epoxidation with different concentrations of Br^-. As shown in the figure, the formation of 1,2-dibromo-2-phenylethane (retention time: 17.8 min) was detected on the chromatogram when the Br^- concentration increased to 5 mM, and it increased with the increase of Br^- concentration.

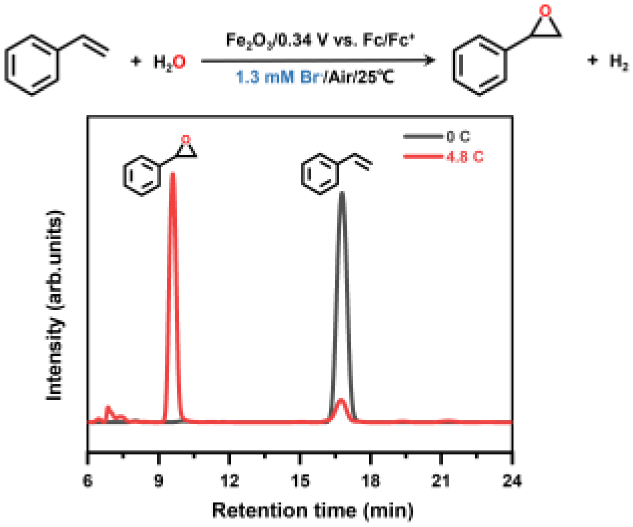

Figure S4. The HPLC spectra of styrene. The PEC reactions were conducted at 0.34 V vs. Fc/Fc^+ for photoelectrocatalysis 4.8 C. The substrate and epoxide product were qualified by the standard curves. The conversion of styrene was 88%, and the corresponding selectivity and FE of epoxide product were 100% and 88%, respectively.

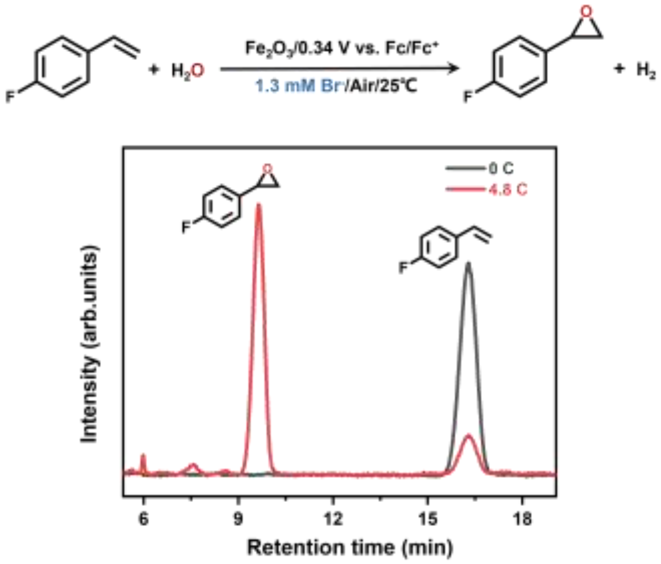

Figure S5. The HPLC spectra of 4-F-styrene. The PEC reactions were conducted at 0.34 V vs. Fc/Fc^+ for photoelectrocatalysis 4.8 C. The substrate and epoxide product were qualified by the standard curves. The conversion of 4-F styrene was 89%, and the corresponding selectivity and FE of epoxide product were 95% and 82%, respectively.

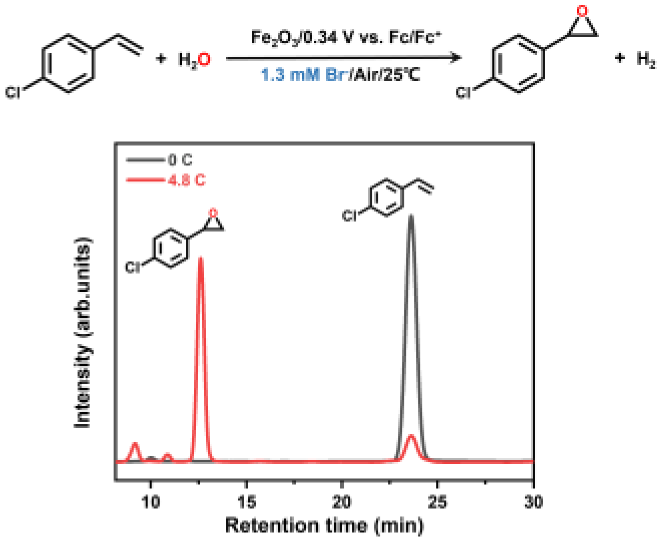

Figure S6. The HPLC spectra of 4-Cl-styrene. The PEC reactions were conducted at 0.34 V vs. Fc/Fc$^+$ photoelectrocatalysis for 4.8 C. The substrate and epoxide product were qualified by the standard curves. The conversion of 4-Cl-styrene was 82%, and the corresponding selectivity and FE of epoxide product were 92% and 82%, respectively.

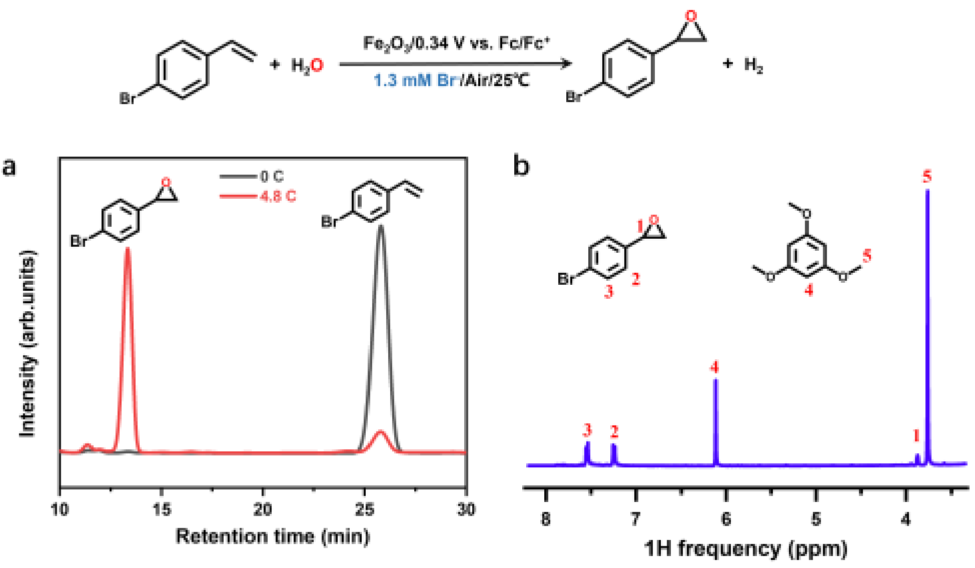

Figure S7. The HPLC spectra a) and the corresponding 1H NMR spectra b) of 4-Br-styrene. The PEC reactions were conducted at 0.34V vs. Fc/Fc^+ for photoelectrocatalysis 4.8 C. An internal standard 1,3,5-trimethoxybenzene (4, 6.12 ppm, s, 3H) was added to quantify the epoxide product (1, 3.86 ppm, t, 1H) in CD_3CN. The conversion of 4-Br-styrene was 95%, and the corresponding selectivity and FE of epoxide product were 95% and 91%, respectively.

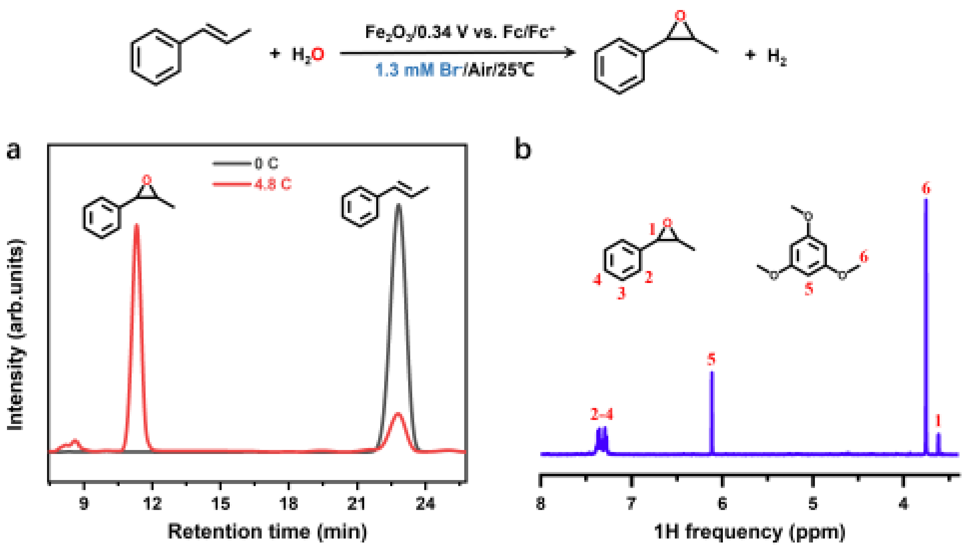

Figure S8. The HPLC spectra a) and the corresponding 1H NMR spectra b) of β-CH_3-styrene. The PEC reactions were conducted at 0.34V vs. Fc/Fc^+ for photoelectrocatalysis 4.8 C. An internal standard 1,3,5-trimethoxybenzene (5, 6.11 ppm, s, 3H) was added to quantify the epoxide product (1, 3.62 ppm, d, 1H) in CD_3CN. The conversion of β-CH_3-styrene was 94%, and the corresponding selectivity and FE of epoxide product were 92% and 86%, respectively.

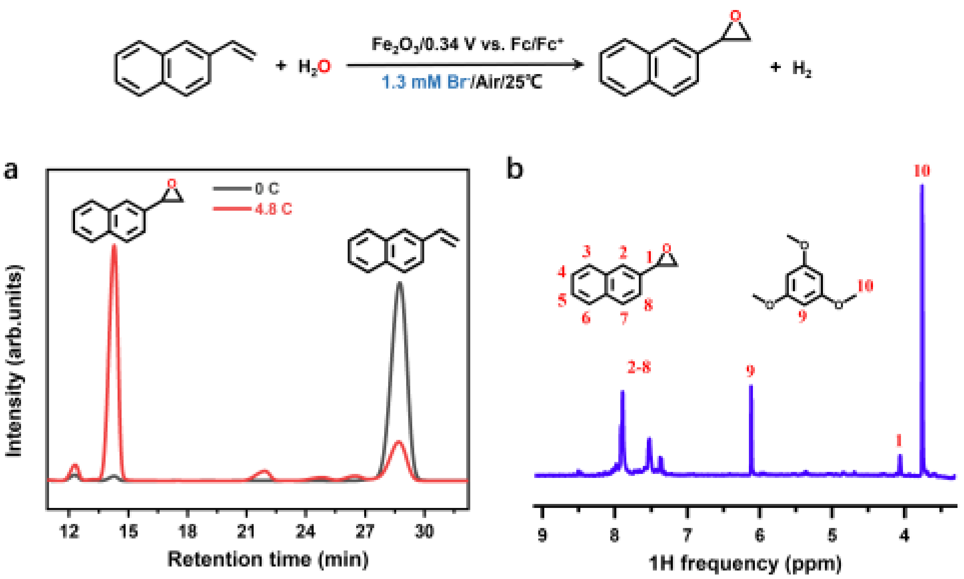

Figure S9. The HPLC spectra a) and the corresponding 1H NMR spectra b) of 2-Vinylnaphthalene. The PEC reactions were conducted at 0.34V vs. Fc/Fc^+ for photoelectrocatalysis 4.8 C. An internal standard 1,3,5-trimethoxybenzene (9, 6.12 ppm, s, 3H) was added to quantify the epoxide product (1, 4.06 ppm, t, 1H) in CD_3CN. The conversion of 2-Vinylnaphthalene was 86%, and the corresponding selectivity and FE of epoxide product were 82% and 71%, respectively.

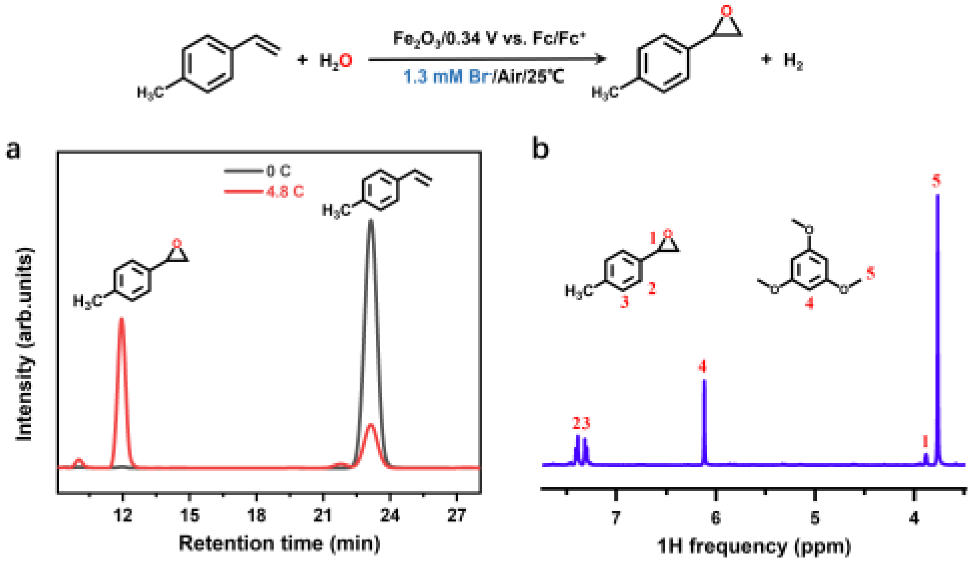

Figure S10. The HPLC spectra a) and the corresponding 1H NMR spectra b) of 4-CH_3-styrene. The PEC reactions were conducted at 0.34V vs. Fc/Fc^+ for photoelectrocatalysis 4.8 C. An internal standard 1,3,5-trimethoxybenzene (4, 6.12 ppm, s, 3H) was added to quantify the epoxide product (1, 3.89 ppm, t, 1H) in CD_3CN. The conversion of 4-CH_3-styrene was 81%, and the corresponding selectivity and FE of epoxide product were 87% and 71%, respectively.

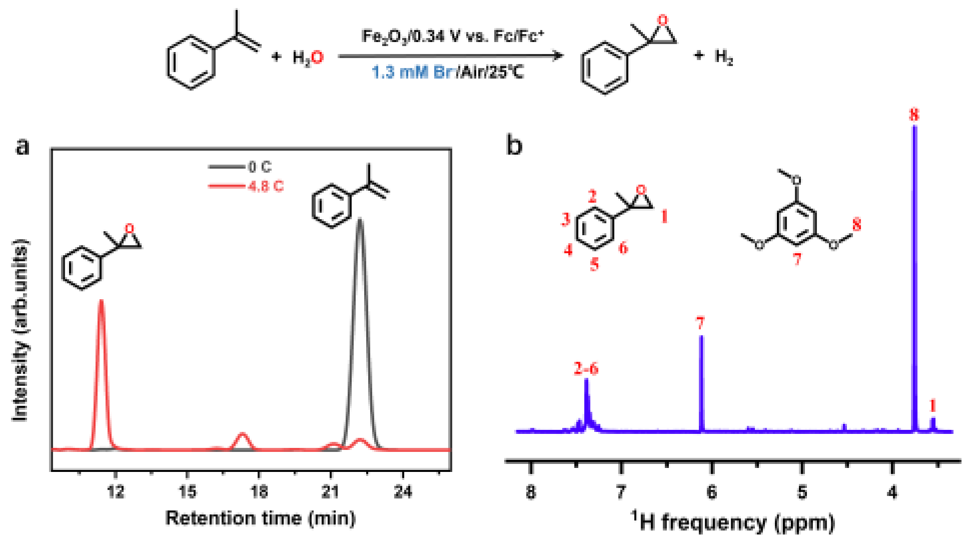

Figure S11. The HPLC spectra a) and the corresponding 1H NMR spectra b) of α-CH_3-styrene. The PEC reactions were conducted at 0.34V vs. Fc/Fc$^+$ for photoelectrocatalysis 4.8 C. An internal standard 1,3,5-trimethoxybenzene (7, 6.12 ppm, s, 3H) was added to quantify the epoxide product (1, 3.56 ppm, d, 1H) in CD_3CN. The conversion of α-CH_3-styrene was 95%, and the corresponding selectivity and FE of epoxide product were 74% and 70%, respectively.

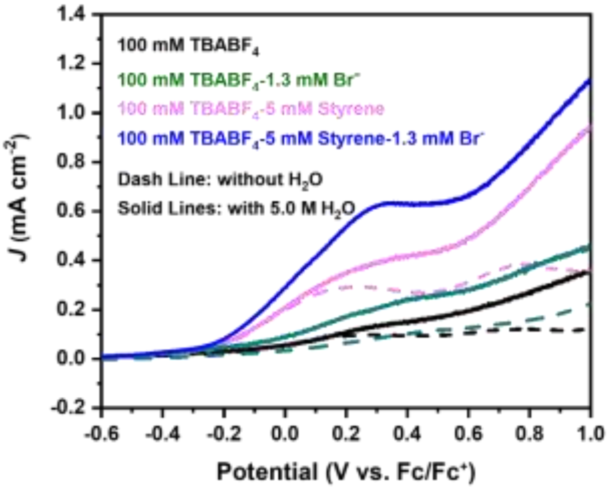

Figure S12. LSVs of the α-Fe_2O_3 photoanode in the 100 mM $TBABF_4$ acetonitrile solution with (solid) or without (dash) water under different conditions. Scan rate, 50 mV s^{-1}

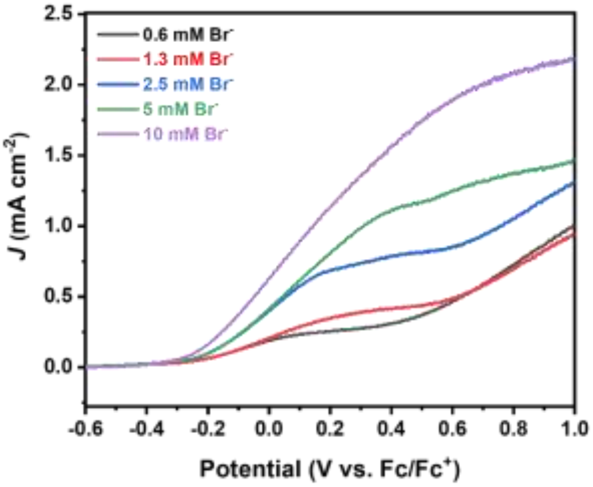

Figure S13. The LSVs of α-Fe_2O_3 with different Br^- concentrations, 5 M H_2O, and 100 mM $TBABF_4$. Scan rate 50 mV s^{-1}.

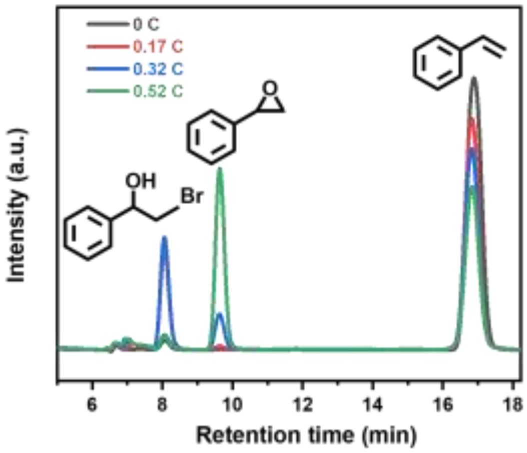

Figure S14. The HPLC spectra obtained at different PEC oxidation coulomb of 1 mM styrene with 100 mM $TBABF_4$ and 1.3 mM Br^- in a one-compartment cell at 0.34 V vs. Fc/Fc^+ on α-Fe_2O_3.

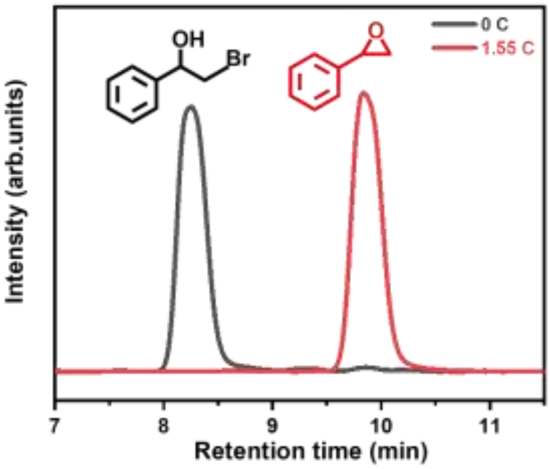

Figure S15 The HPLC spectra of photoelectrolytic experiment in the two-compartment cell with 1 mM bromohydrin added in the cathodic cell. Reaction conditions: 1.3 mM Br^-, 100 mM $TBABF_4$, 5 M H_2O, 1000 rpm, and 16 mL total reaction solution.

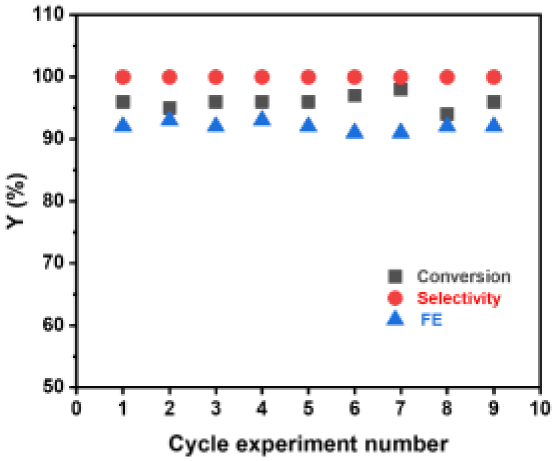

Figure S16. The photoelectrolytic epoxide results of the α-Fe_2O_3 cycle experiments in 1 mM styrene, 5 M H_2O and 0.1 M $TBABF_4$.

The nine cycles experiments of styrene epoxidation show that the conversion of styrene is maintained at ~ 95%, and the selectivity and FEs of epoxide are maintained at ~ 100% and ~ 92%, respectively. The above results indicate that the α-Fe_2O_3 has good photoelectrochemical stability.

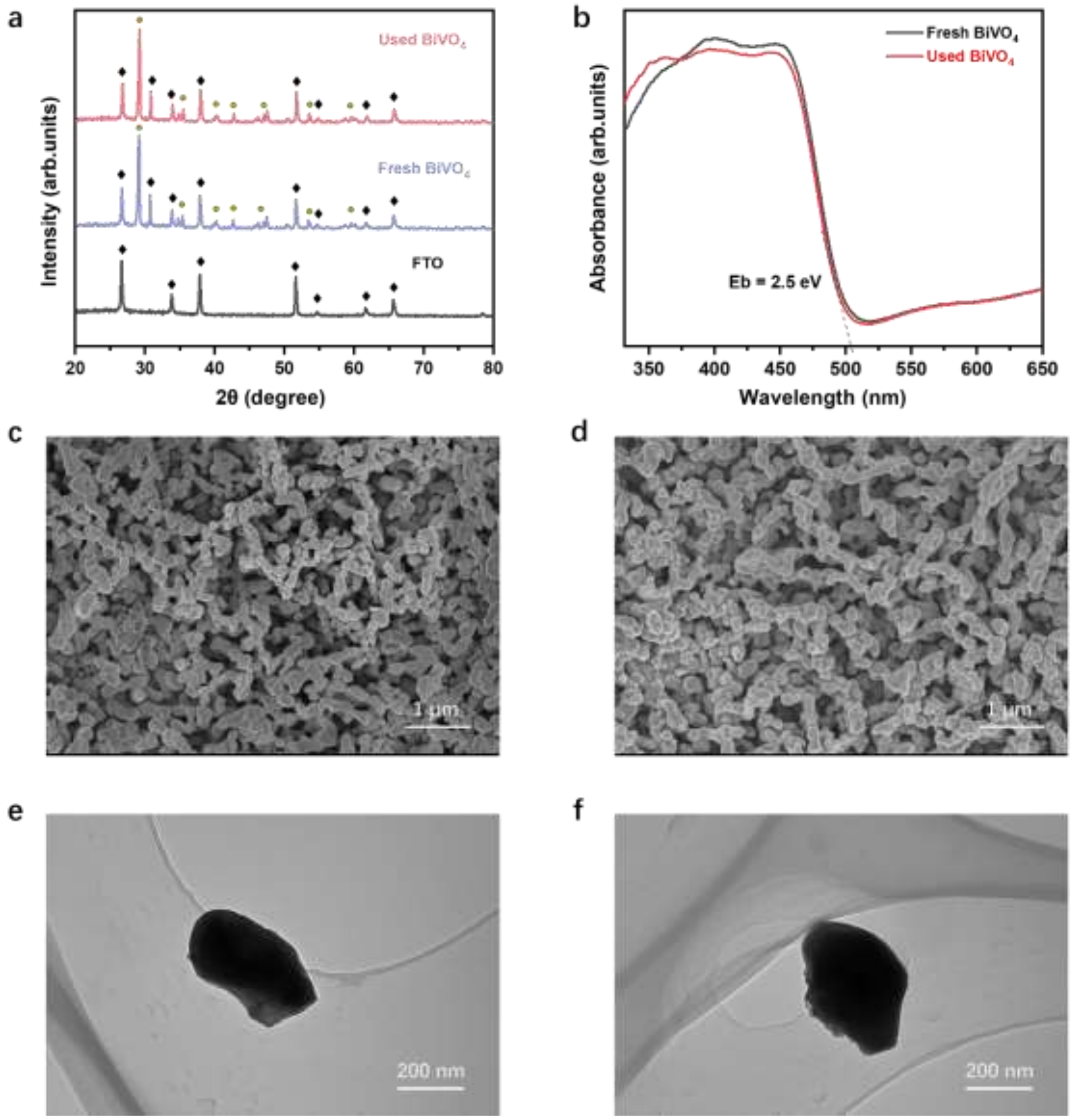

Figure S17. The characterizations of fresh and used $BiVO_4$ photoanodes. a) XRD spectra; b) UV-vis diffuse spectra; SEM images of fresh c) and used d) $BiVO_4$; TEM images of fresh e) and used f) $BiVO_4$.

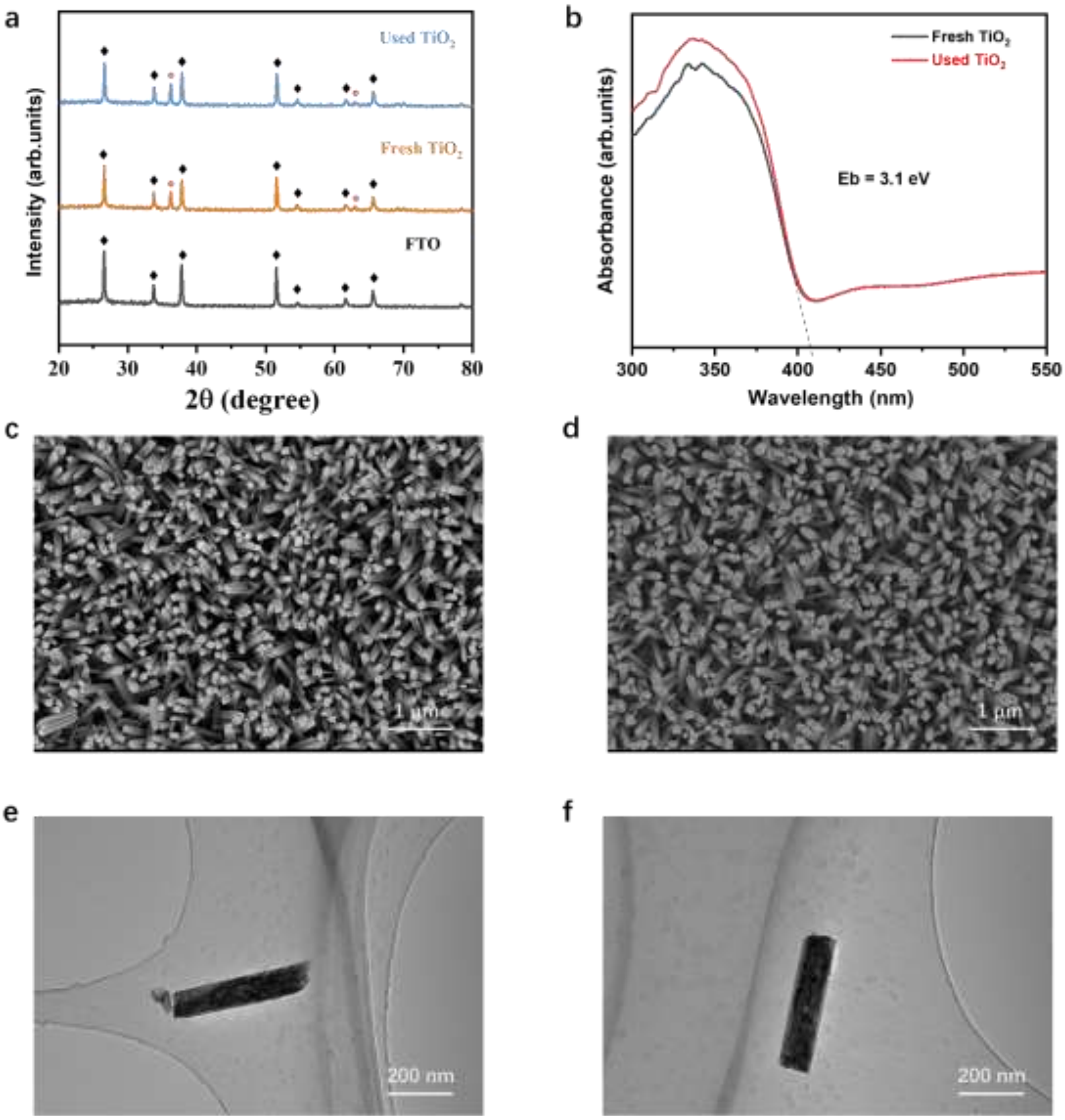

Figure S18. The characterizations of fresh and used TiO_2 photoanodes. a) XRD spectra; b) UV-vis diffuse spectra; SEM images of fresh c) and used d) TiO_2; TEM images of fresh e) and used f) TiO_2.

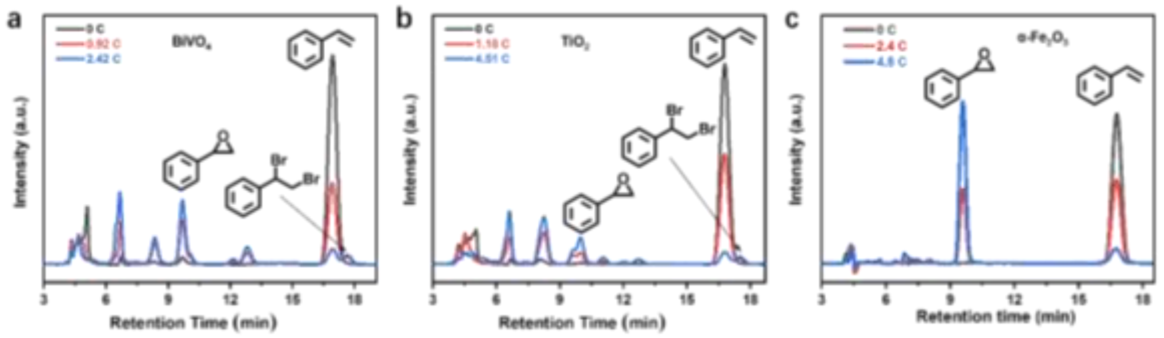

Figure S19. The HPLC spectrum of styrene epoxidation with a) $BiVO_4$, b) TiO_2 and c) α-Fe_2O_3 photoanodes.

The epoxidation of styrene was under the condition of Air atmosphere, 0 rpm, 1 mM styrene, 5 M H_2O and 100 mM $TBABF_4$. In the almost complete conversion of styrene substrate for $BiVO_4$ and TiO_2 photoanode, the selectivity of epoxy product was 30% and 4% respectively, FEs were as low as 11% and 1%, respectively. The above data show that the selectivity and FE of styrene epoxidation are increased to 99% and 88% due to the oxygen atom transfer characteristics of the α-Fe_2O_3 photoanode.

The significant 1,2-dibromo-2-phenylethane by-product is observed in the $BiVO_4$ and TiO_2 system (Figure S18a and b). The formation of 1,2-dibromo-2-phenylethane should step from Br_2 produced in $BiVO_4$ and TiO_2 system. By contrast, the selectivity for epoxide is quite high (~ 100%) and almost no dibrominated product is formed on α-Fe_2O_3 under otherwise identical conditions.

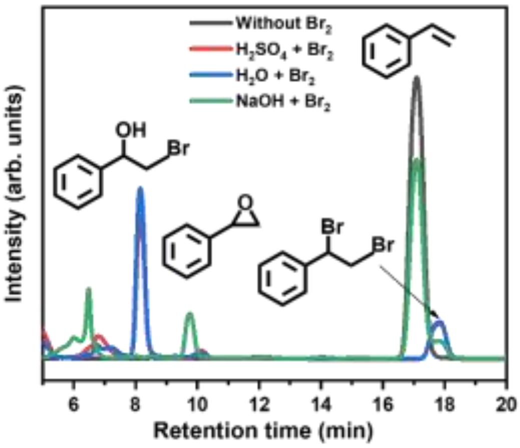

Figure S20. The HPLC spectra of the reaction between styrene and Br_2 by adding Br_2 into the CH_3CN solution with 5 M of water at different pHs.

Br_2 molecule (0.5 M, 256 µL) was added into 10 mL H_2SO_4 aqueous solution with pH=1, 10 mL H_2O, and 10 mL NaOH aqueous solution with pH=13, respectively. Then the mixed solution was stirred for 20 min under ice-bath condition and the obtained solution (0.9 mL) was respectively transferred into 9.1 mL CH_3CN with stirring under ice-bath condition to obtain 50 mM Br_2 of CH_3CN solution with 5 M of water at different pHs. Finally, 1 mL 50 mM Br_2 of CH_3CN solution with 5 M of water at different pHs and 1 mL 10 mM styrene solution were mixed and stirred thoroughly, and the mixed solution was tested by HPLC. As shown in Figure S19, 1,2-dibromo-2-phenylethane is formed regardless of the acidity and basicity.

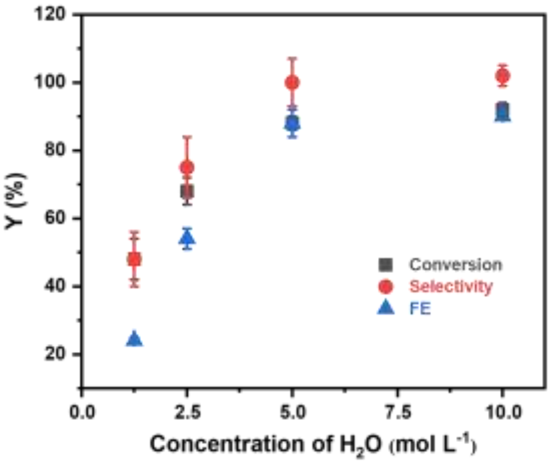

Figure S21. The epoxidation performance for different concentrations (1.25 ~10 M) of H_2O at 0.34 V vs. Fc/Fc^+ with 1.3 mM Br^-. Each error bar denotes the standard deviation of data from three experiments.

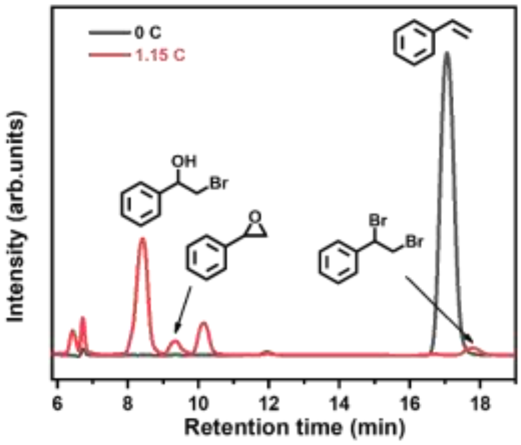

Figure S22. The HPLC spectra of before and after reaction with 1.3 mM Br^-, 1.25 M H_2O and 16 mL total solution in the two-compartment cell.

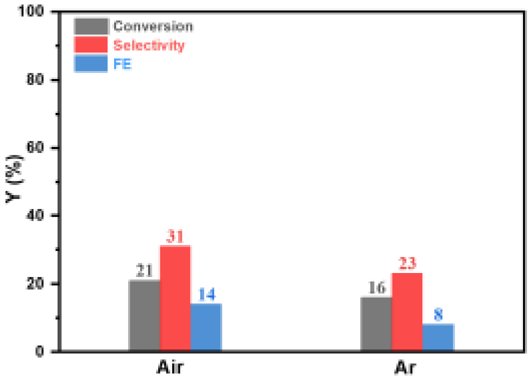

Figure S23. The epoxidation performance of 1.25 M H_2O, 1.3 mM Br^- and 5 mM styrene system in different reaction atmospheres of a single cell. The results suggest that oxygen is not responsible for the low selectivity at 1.25 M H_2O.

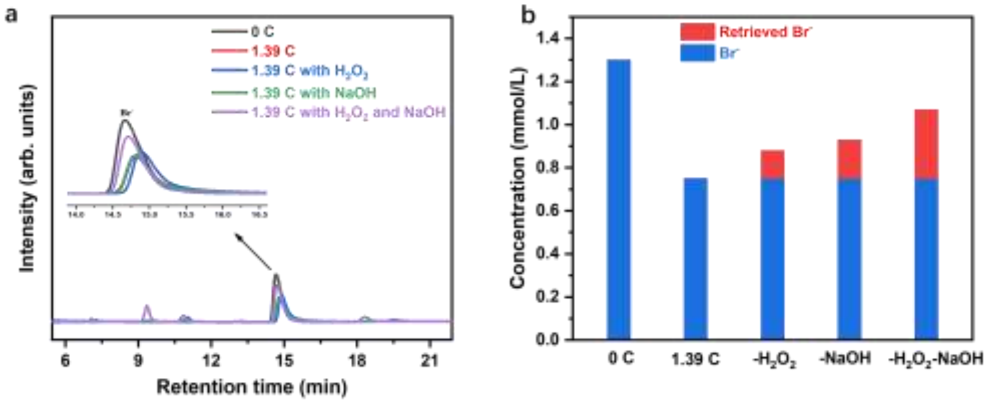

Figure S24. The IC spectra a) and the corresponding Br^- concentration b) of reaction solution with electrolysis through 1.39 C before and after in the two-compartment cell. The Br^- are recovered by adding trace amounts of H_2O_2 or (and) NaOH to the reaction solution. The final recovery ratio of Br^- is up to 82%, suggesting the lost Br^- is present in the solution as inorganic and organic bromine.

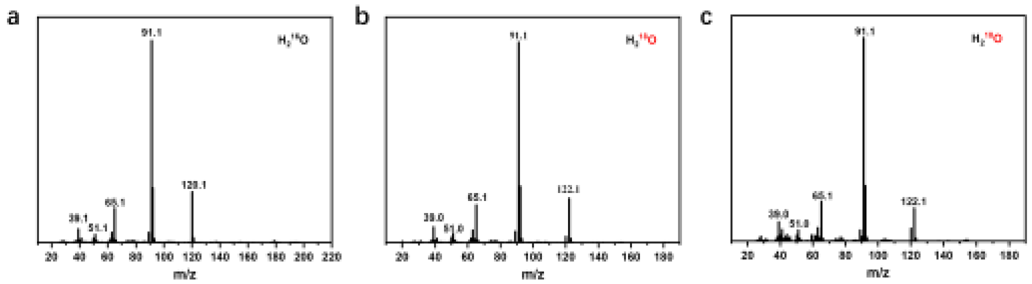

Figure S25. Mass spectra of the styrene oxide during the oxidation of the styrene with $H_2^{16}O$ a) or $H_2^{18}O$ b) in the single cell and $H_2^{18}O$ c) in the H cell, where bromohydrin is converted to styrene oxide by adding 5 M NaOH solution.

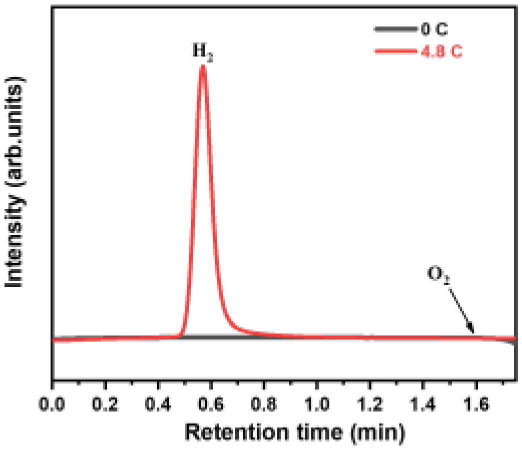

Figure S26. The GC spectra of the headspace gas of the PEC cell after 4.8 C photoelectrolysis. The Faradaic Efficiency of the hydrogen evolution was about 95%. No dioxygen (~ 1.6 min) was detected in GC spectra, which indicates that water oxidation to O_2 rarely occurs in the low concentration Br^- mediated PEC epoxidation.

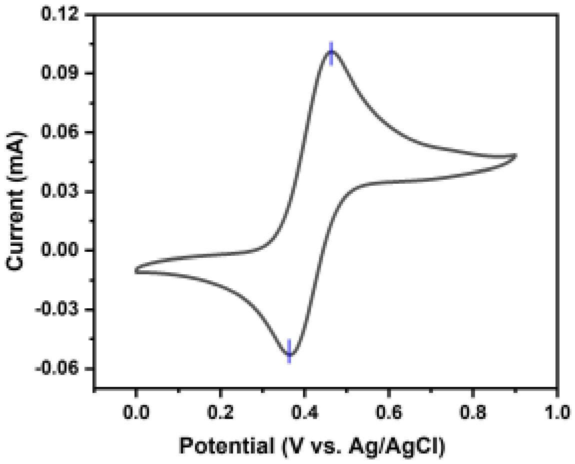

Figure S27. Cyclic voltammetry curves of 5 mM ferrocene solution by a glassy carbon electrode in CH_3CN with 5 M H_2O in the presence of 0.1 M $TBABF_4$ electrolyte (scan rate: 0.05 V s^{-1}). $E_{1/2}$ = 0.46 V vs. Fc/Fc^+.

Accordingly, the applied potential was calibrated according to the following equation:

E(vs. Fc/Fc^+) = E(vs. Ag/AgCl) - 0.46 V.

Table S1. Examples with the best performance for previously reported PEC or EC styrene epoxidation.

Entry	Substrate	anode	mediator	Select. (%)	FE (%)	Yield (%)	Ref.
1[a)]	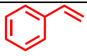	PEC on α-Fe_2O_3	0.26 eq Br^-	100	88	88	This work
2[b)]	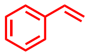	PEC on α-Fe_2O_3	0.26 eq Br^-	100	~100	80	This work
3	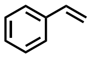	PEC on α-Fe_2O_3	10 eq TBABr	95	34	48	[2]
4	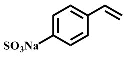	PEC on $BiVO_4$	1 eq NaBr	>99	65	>99	[3]
5	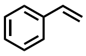	EC on GF-CoS_2/ CoS	2 eq NaBr	97	-	97	[4]
6	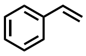	EC on Pt	1 eq NaBr	79	30	79	[5]
7	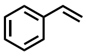	EC on Pt	1.5 eq NaBr	72	-	66	[6]
8	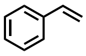	EC on Graphite	0.25 eq NaBr	83	40	71	[7]
9		EC on Pt foil	KCl	97	70	-	[8]
10	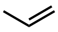	PEC on CoPi/$BiVO_4$	no	98	-	-	[9]
11	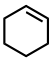	PEC on α-Fe_2O_3	no	-	35±2	72±4	[10]
12	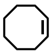	EC on Mn_3O_4	no	72	30	28	[11]
13	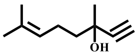	EC on Pt foil	NaBr	77	33	77	[12]
14	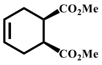	EC on Pt	NaBr	97	39	-	[13]
15	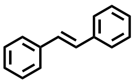	EC on CoS_2-CoS-GF	2 eq NaBr	99	14	40	[4]

[a)] The one-compartment cell, 5 mL solution containing 5 mM styrene in CH_3CN (5 M H_2O) with 1.3 mM Br^- and 100 mM $TBABF_4$, room temperature, applied potential 0.34 V vs. Fc/Fc^+.

[b)] The two-compartment cell, total solution 16 mL containing 1 mM styrene in CH_3CN (5 M H_2O) with 1.3 mM Br^- and 100 mM $TBABF_4$, room temperature, applied potential 0.34 V vs. Fc/Fc^+, 20 µL 1 M H_2SO_4 was added to the anode chamber.

The entry 10: A titanium silicalite-1 heterogeneous catalyst was used to epoxidize the propylene directly.

Table S2. The concentration of Br^- and BrO_3^- before and after the PEC low concentration Br-mediated styrene epoxidation reaction in the one-compartment cell of different stirring speeds with 5 M H_2O and 5 mM styrene at 0.34 V vs. Fc/ Fc^+ on α-Fe_2O_3.

Entry	The stirring speed	The PEC coulomb	The concentration of Br^-	The concentration of BrO_3^-	The FE of BrO_3^-
1	0 rpm	0 C	1.24 mM	0 mM	0%
2	0 rpm	4.8 C	1.06 mM	0.16 mM	9%
3	1000 rpm	4.8 C	0.74 mM	0.18 mM	11%

Table S3. The concentration of Br^- and BrO_3^- before and after the PEC low concentration Br^-mediated styrene epoxidation reaction in the one-compartment cell of different H_2O content speed with 0 rpm stirring speed and 5 mM styrene at 0.34 V vs. Fc/ Fc^+ on α-Fe_2O_3.

Entry	The water content	The PEC coulomb	The concentration of Br^-	The concentration of BrO_3^-	The FE of BrO_3^-
1	1.25 M	0 C	1.25 mM	0 mM	0%
2	2.5 M	0 C	1.22 mM	0 mM	0%
3	5 M	0 C	1.22 mM	0 mM	0%
4	1.25 M	4.8 C	0.56 mM	0.06 mM	4%
5	2.5 M	4.8 C	0.72 mM	0.09 mM	7%
6	5 M	4.8 C	1.06 mM	0.16 mM	9%

References

[1] Y. Zhao, M. Duan, C. Deng, J. Yang, S. Yang, Y. Zhang, H. Sheng, Y. Li, C. Chen, J. Zhao, *Nat. Com.* **2023**, *14*, 1943.

[2] X. Liu, Z. Chen, S. Xu, G. Liu, Y. Zhu, X. Yu, L. Sun, F. Li, *J. Am. Chem. Soc.* **2022**, 144, *43*, 19770.

[3] Y. Zhang, A. Iqbal, J. Zai, S.-Y. Zhang, H. Guo, X. Liu, I. ul Islam, H. Fazal, X. Qian, *Org. Chem. Front.* **2022**, *9*, 436.

[4] M. Inês, A. J. Mendonça, A. P. Esteves, D. I. Mendonça, M. J. Medeiros, *C R Chim.* **2009**, *12*, 841.

[5] H. Tang, J. R. Vanhoof, D. De Vos, *Green Chem.* **2022**, *24*, 9565.

[6] W. Jud, C. O. Kappe, D. Cantillo, *Electrochem. Sci. Adv.* **2021**, *1*, e2100002.

[7] W. R. Leow, Y. Lum, A. Ozden, Y. Wang, D.-H. Nam, B. Chen, J. Wicks, T.-T. Zhuang, F. Li, D. Sinton, E. H. Sargent, *Science* **2020**, *368*, 1228.

[8] M. Ko, Y. Kim, J. Woo, B. Lee, R. Mehrotra, P. Sharma, J. Kim, S. W. Hwang, H. Y. Jeong, H. Lim, S. H. Joo, J. W. Jang, J. H. Kwak, *Nat. Catal.* **2022**, *5*, 37.

[9] M. Tayebi, Z. Masoumi, A. Tayyebi, J.-H. Kim, H. Lee, B. Seo, C.-S. Lim, H.-G. Kim, *ACS Appl. Mater. Inter.* **2023**, *15*, 20053.

[10] K. Jin, J. H. Maalouf, N. Lazouski, N. Corbin, D. Yang, K. Manthiram, *J. Am. Chem. Soc.* **2019**, *141*, 6413.

[11] S. Torii, K. Uneyama, M. Ono, H. Tazawa, S. Matsunami, *Tetrahedron Lett.* **1979**, *20*, 4661.

[12] S. Torii, K. Uneyama, H. Tanaka, T. Yamanaka, T. Yasuda, M. Ono, Y. Kohmoto, *J. Org. Chem.* **1981**, *46*, 3312.
